# An “UninTENSional” Subcutaneous Implantable Cardioverter-defibrillator Shock

**DOI:** 10.19102/icrm.2018.091106

**Published:** 2018-11-15

**Authors:** Suzette L. Turner, Sheldon M. Singh

**Affiliations:** ^1^Department of Cardiology, Sunnybrook Health Sciences Centre, Toronto, ON, Canada

**Keywords:** Electromagnetic interference (EMI), inappropriate shock, subcutaneous implantable cardioverter-defibrillator

## Abstract

Subcutaneous implantable cardioverter-defibrillators (ICDs) (S-ICDs) are advantageous because they eliminate the need for transvenous leads. However, just like in the case of traditional ICDs, inappropriate shocks are an unwanted complication that may result following their placement. In this case, we discuss the mechanism of an inappropriate shock in a patient with an S-ICD.

## Introduction

Subcutaneous implantable cardioverter-defibrillators (ICDs) (S-ICDs) are considered by many to be advantageous over more traditional ICDs due to their elimination of the need for transvenous leads. However, with either type of device, inappropriate shock can occur as an unwanted complication. The Boston Scientific Postmarket S-ICD Registry (EFFORTLESS) noted that 8.3% of its 581 patients with S-ICDs had inappropriate shocks (average of 2.2 shocks per patient). This translated to 48 patients over a period of 21 months ± 13 months, with the registry beginning in 2009.^1^ In the current case, we discuss the presentation of an S-ICD patient who used a transcutaneous electrical nerve stimulation (TENS) device and suffered an inappropriate shock. This case highlights a limitation of long-term S-ICD sensing.

## Case presentation

A 41-year-old female with nonischemic cardiomyopathy received a model 1010 SQ-RX S-ICD (Boston Scientific, Natick, MA, USA). Adequate QRS sensing was present at the time of implantation and during ventricular fibrillation (VF) induction. The secondary sensing configuration was used and VF therapy was programmed to 230 beats per minute (bpm) with a conditional zone of 200 bpm.

**Figure 1:**
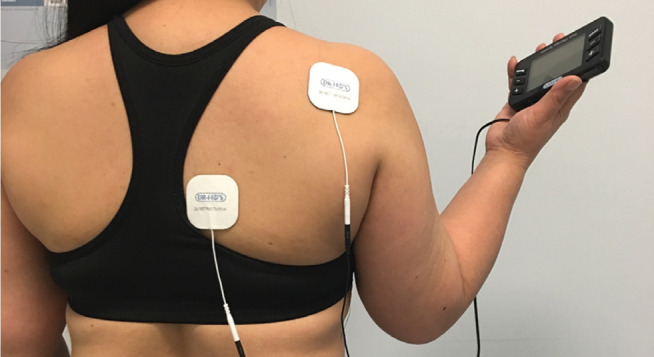
The TENS massager used by the patient in the present case.

Three-and-a-half years after implantation, the patient experienced an ICD shock while using a TENS unit on her neck, axilla, and back **(Figure 1)**. The patient stated that she had frequently used her massager without issue prior to this episode.

**Figure 2:**
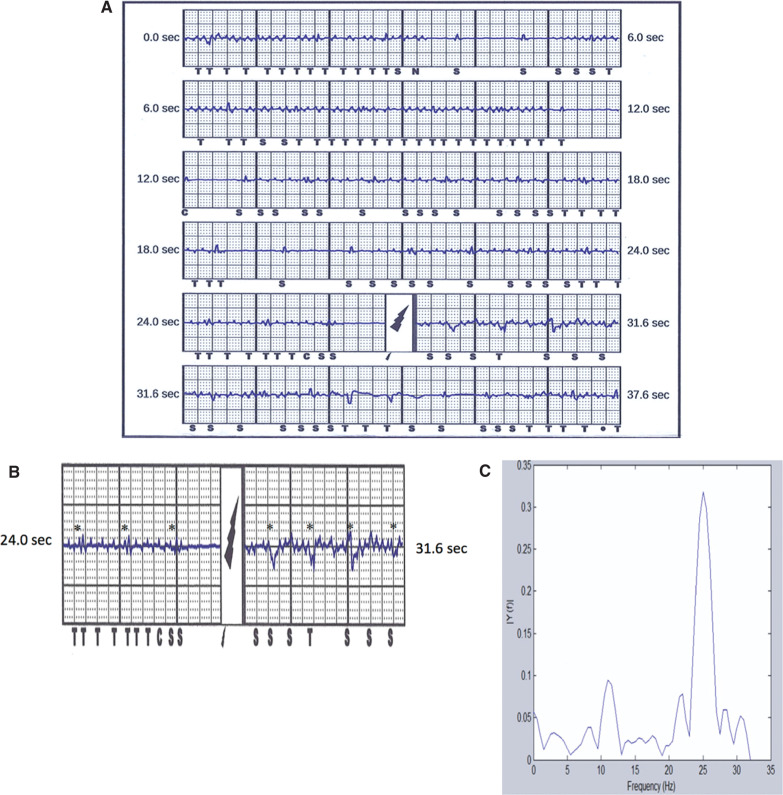
Treated episodes. **A:** Shock episode. **B:** Noise episode magnified. **C:** Spectral analysis of noise.

A review of the electrograms demonstrated a low-amplitude QRS **(Figures 2A–2C)**. Additional low-amplitude, high-frequency signals with amplitudes of 0.35 mV to 0.5 mV were noted during the event and sensed by the patient’s S-ICD as intrinsic cardiac electrical activity. As the heart rate perceived by this patient’s device was consistent with VF, an ICD shock was administered. Closer examination of the event clearly demonstrated that the patient’s native QRS **(Figure 2B)** was present during, and independent of, these high-frequency signals, thereby confirming the presence of noise.

**Figure 3:**
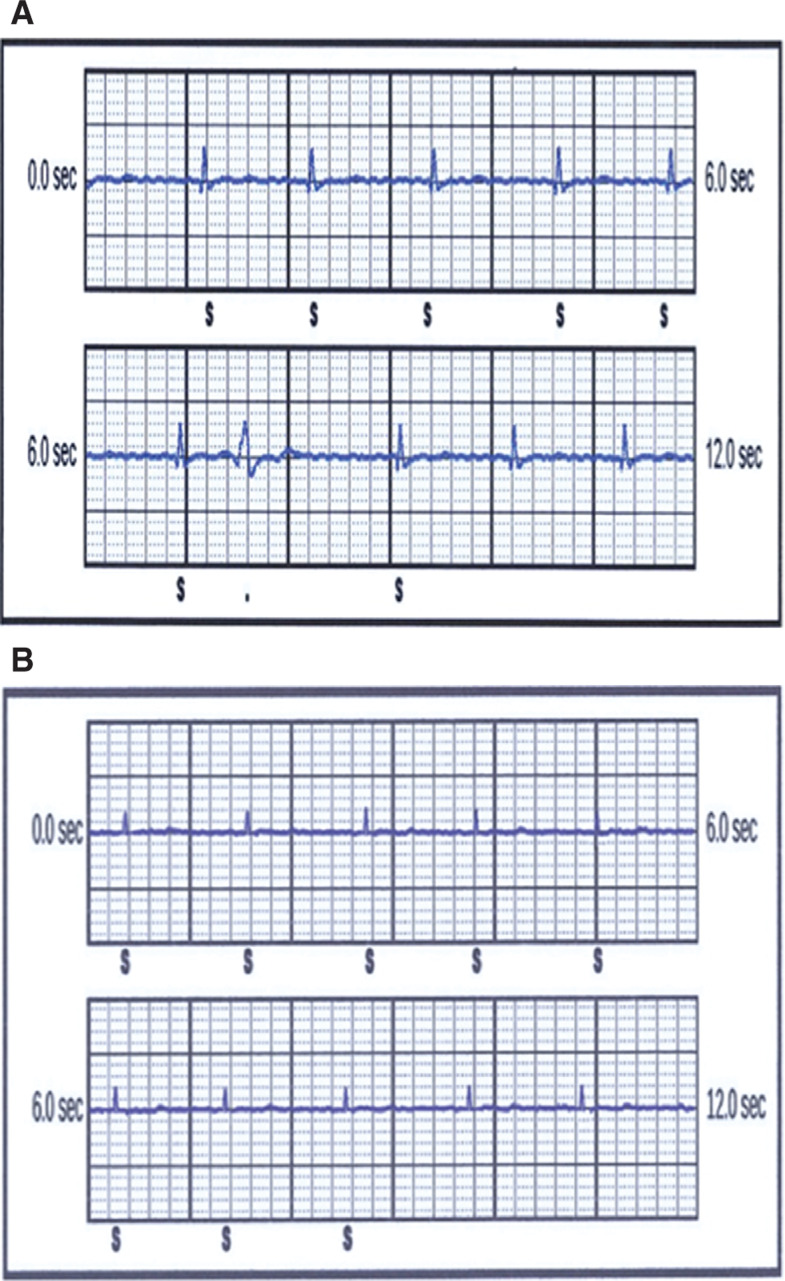
Patient electrocardiograms (ECGs). **A:** ECG at S-ICD implant. **B:** ECG at 3.5 years after S-ICD implant.

External analysis of this event demonstrated a single dominant frequency consistent with electromagnetic interference (EMI) **(Figure 2C)** that was temporally related to the use of the modulated TENS device. The patient was advised not to use the TENS device again. The QRS noted at implant was 1.6 mV **(Figure 3A)** and the true QRS at the time of the shock episode was 0.8 mV **(Figure 3B)**. Although her QRS was now lower than at the time of implantation, as sensing was stable and similar to other vectors, we did not make additional device changes.

We speculate that the combination of a low-amplitude native QRS with external noise resulted in inappropriate VF detection and delivery of therapy. The progressive decrease in our patient’s QRS voltage with time may possibly explain the timing of her inappropriate shock, which occurred for the first time at 3.5 years after S-ICD implantation, despite ongoing use of the same TENS device since the time of her S-ICD implant procedure.

## Discussion

EMI is an electrical signal generated by an internal or external source that falls within the sensing frequency spectrum of an implantable cardiac electronic device that may result in, or trigger, inappropriate device behavior. Although sensing algorithms and low-pass filters within implantable devices are designed to mitigate inappropriate device behaviors, they are not infallible. One may speculate that the sensing vectors in the S-ICD created by the superficial location and wide antennae of the device can increase its susceptibility to EMI and myopotential oversensing as compared with transvenous ICDs. In our case, the low sensed QRS amplitude may have contributed to the inability to filter out the external noise, as the estimated starting peak for the refractory period would be reduced, allowing for a lower or more sensitive absolute floor for detection.

Offline spectral analysis of the event was invaluable, as it confirmed EMI rather than myopotential oversensing. The latter is another rare cause of inappropriate therapy deliverance in S-ICD patients.^2^ In the former situation, one may simply instruct patients on the need to avoid the device causing the EMI, whereas the latter may require additional device reprogramming or repositioning.

While the use of TENS is contraindicated in patients with ICDs, patients may still utilize this therapy despite recommendations. Indeed, our case is not unique, as there are prior reports of inappropriate shocks due to TENS use in transvenous ICD patients.^3–6^ However, to our knowledge only one report of an inappropriate shock due to TENS use has been reported before in an S-ICD patient.^7^ Our case highlights the potential impact of long-term changes in device sensing on the delivery of ICD therapy in special situations, such as the presence of EMI. This case should remind clinicians of the limitations of ICD sensing and promote patient education on day-to-day activities that may adversely impact their ICDs.
